# The RNA Polymerase-Associated Factor 1 Complex Is Required for Plant Touch Responses

**DOI:** 10.1093/jxb/erw439

**Published:** 2016-12-15

**Authors:** Gregory S. Jensen, Kateryna Fal, Olivier Hamant, Elizabeth S. Haswell

**Affiliations:** 1 Department of Biology, Mailbox 1137, Washington University in Saint Louis, Saint Louis, MO 63130, USA; 2 Current address: Donald Danforth Plant Science Center, 975 North Warson Road, St. Louis, MO 63132, USA; 3 Laboratoire Reproduction et Développement des Plantes, Univ Lyon, ENS de Lyon, UCB Lyon 1, CNRS, INRA, F-69342, Lyon, France

**Keywords:** Histone methylation, Paf1 complex, *TCH* genes, thigmomorphogenesis, touch response, *VIP3*.

## Abstract

Thigmomorphogenesis is a stereotypical developmental alteration in the plant body plan that can be induced by repeatedly touching plant organs. To unravel how plants sense and record multiple touch stimuli we performed a novel forward genetic screen based on the development of a shorter stem in response to repetitive touch. The *touch insensitive (ths1*) mutant identified in this screen is defective in some aspects of shoot and root thigmomorphogenesis. The *ths1* mutant is an intermediate loss-of-function allele of *VERNALIZATION INDEPENDENCE 3* (*VIP3*), a previously characterized gene whose product is part of the RNA polymerase II-associated factor 1 (Paf1) complex. The Paf1 complex is found in yeast, plants and animals, and has been implicated in histone modification and RNA processing. Several components of the Paf1 complex are required for reduced stem height in response to touch and normal root slanting and coiling responses. Global levels of histone H3K36 trimethylation are reduced in *VIP3* mutants. In addition, *THS1/VIP3* is required for wild type histone H3K36 trimethylation at the *TOUCH3* (*TCH3*) and *TOUCH4* (*TCH4*) loci and for rapid touch-induced upregulation of *TCH3* and *TCH4* transcripts. Thus, an evolutionarily conserved chromatin-modifying complex is required for both short- and long-term responses to mechanical stimulation, providing insight into how plants record mechanical signals for thigmomorphogenesis.

## HIGHLIGHT

The chromatin-modifying complex Paf1 is required for shoot and root thigmomorphogenesis and touch-inducible gene expression.

## Introduction

It is firmly established that development in multicellular organisms relies on the local concentration of key biochemical signals such as hormones or growth factors (Wolpert, 1969). However, it is becoming increasingly clear that the temporal pattern of changes in the concentration of these biochemical signals is equally important for the final phenotypic output (Raj and van Oudenaarden, 2008; Lander, 2011; Oates, 2011; Webb *et al.*, 2016). For instance, the intrinsic delays associated with transcription and translation have been proposed to play an instructive role in patterning (Monk, 2003; Gaffney and Monk, 2006). Understanding how the intensity as well as the frequency of a stimulus are measured and recorded is a key challenge for plant biologists, as post-embryonic development is continuously impacted by the environmental conditions in which the plant grows.

Mechanical stimuli are coded both in intensity and in frequency [for examples see (Chehab *et al.*, 2009; Leblanc-Fournier *et al.*, 2014)] but how this is accomplished at the molecular and genetic level is unknown. Here we begin to address the genetic pathways by which a repeated mechanical stimulus leads to a developmental change during plant thigmomorphogenesis, a stereotypical developmental alteration in the plant body plan that can be induced by shaking, rubbing, bending, or brushing leaves or stems (Chehab *et al.*, 2009; Coutand, 2010). Thigmomorphogenesis is classically defined as producing a shorter and thicker plant due to decreased stem height and increased stem and/or petiole diameter relative to control plants (Jaffe, 1980; Biddington, 1986; Chehab *et al.*, 2009; Coutand, 2010). Repeated mechanical stimulation can also result in a more flexible stem and an increase in root mass relative to the shoot. Taken together, these morphological changes are thought to produce a body plan that is more resistant to damage upon future mechanical challenges. This presents an intriguing conversion of energy from an elastic deformation (i.e. bending) into a developmental response (i.e. growth).

Three hallmarks of thigmomorphogenesis are: a dose responsiveness that prevents the plant from responding to isolated events, a systemic nature such that a stimulus applied to one organ leads to changes in overall plant morphology, and a characteristic delay between the initial stimulus and the developmental response (Chehab *et al.*, 2009; Leblanc-Fournier *et al.*, 2014; Moulia *et al.*, 2015). The mechanical stimulus received by cells in the rosette leaves of plants that have been touched, sprayed, blown or bent must be transmitted to the cells of the shoot apical meristem in order to control elongation of the future shoot or stem (Moulia *et al.*, 2015).

Indeed, there is evidence that plants are able to record the number and frequency of mechanical stimuli. The Venus flytrap uses action potentials to measure the number of times an insect touches the trigger hairs of its trap over the course of an hour (Böhm *et al.*, 2016). Plants are also able to sense the number and frequency of stem bending events. When several bends are imposed on poplar stems, the impact on the transcriptome becomes less pronounced from the second bending event onwards. It also takes no less than a week without bending for a stem to recover full molecular sensitivity to such mechanical deformation (Martin *et al.*, 2010). Thus, plants are indeed capable of recording successive mechanical deformations.

Plants also respond to touch in the short-term, though how these events are connected to long-term changes in body plan remains unclear. Following a single touch event, plants rapidly upregulate a large number of genes; in Arabidopsis as much as 2.5% of its genome (Braam and Davis, 1990; Lee *et al.*, 2005). The best characterized of these in Arabidopsis are the *TOUCH* (*TCH*) genes, which encode calmodulins and calmodulin-like proteins, cell wall-modifying enzymes, and wound- and defense-inducible genes (Braam and Davis, 1990; Ling *et al.*, 1991; Perera and Zielinski, 1992; Kagaya and Hattori, 2009; Lee *et al.*, 2005). The timing of the induction of most *TCH* genes is between 5 and 30 minutes after the application of stimuli, suggesting a role in the early response to touch stimulation. The *cml24 (tch2*) mutation affects root morphology on hard agar and is implicated in microtubule structure and starvation-induced autophagy (Wang *et al.*, 2011; Tsai *et al.*, 2013) but a functional role for *TCH* genes in thigmomorphogenesis has yet to be established. While these results demonstrate that touch is perceived at the molecular level after a single event, no link between short-term and long-term responses to touch has been identified. In trees, the transcription factor gene *ZFP2* is rapidly induced in response to bending (Leblanc-Fournier *et al.*, 2008; Coutand *et al.*, 2009). Interestingly, this induction can be attenuated after multiple bending events, suggesting that plants are able to record the number of mechanical perturbations, albeit through an unknown mechanism (Martin *et al.*, 2010).

The growth response is likely to involve the phytohormone jasmonic acid (JA). JA accumulates in the stems of plants after touching of their rosette leaves, and JA biosynthesis and signaling are required for thigmomorphogenesis (Chehab *et al.*, 2012). Conversely, application of the growth regulatory hormone gibberellin or a loss-of-function mutation in the gibberellin-catabolizing enzyme AtGA2ox7 can prevent thigmomorphogenesis (Lange and Lange, 2015). However, these effects are potentially far downstream in the pathway. No real known regulator of thigmomorphogenesis capable of sensing repetition has been identified yet.

We therefore performed a novel forward genetic screen designed to identify the key molecular and genetic components that link successive elastic deformations to thigmomorphogenesis. Arabidopsis exhibits several stereotypical thigmomorphogenic responses that require multiple days of touch, including the inhibition of stem elongation, shortened petioles, reduced rosette diameter and delayed transition to flowering (Braam and Davis, 1990; Chehab *et al.*, 2012; Cazzonelli *et al.*, 2014; Lange and Lange, 2015). Here we concentrated on stem thigmomorphogenesis to identify new regulators of the touch response. We screened for plants that did not exhibit shortened stems after repeated touch. The first mutant isolated in this screen identified a known regulator of gene expression, the RNA Polymerase II-associated factor 1 (Paf1) complex, as a key element of thigmomorphogenesis that serves to integrate touch stimuli over time.

## Materials and methods

### Touch response assays

**Fig. 1.**
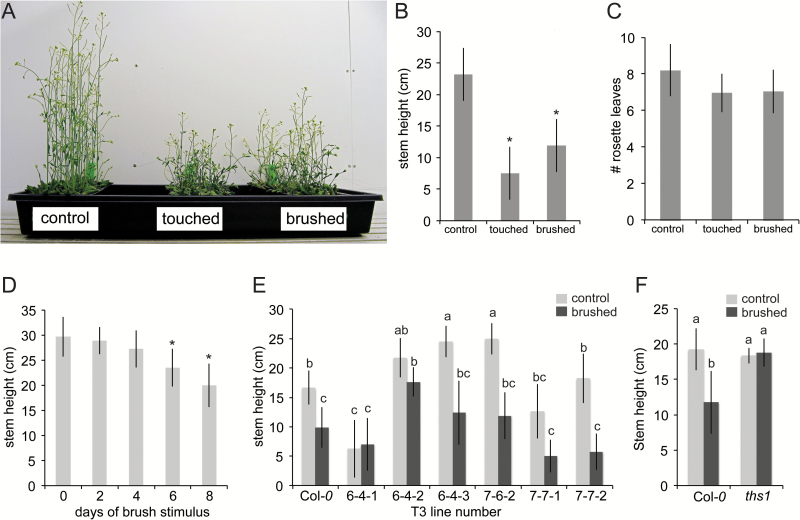
**A forward genetic screen identifies an Arabidopsis mutant insensitive to repeated touch.** (A-C) Characterization of hand touch-induced and paintbrush-induced thigmomorphogenesis. Two-week-old Col*-0* seedlings grown on soil were either untreated, touched by a gloved hand or brushed with a paintbrush (10 passes per day). Plants were then grown for an additional 10 days without stimulus. (A) Image of control, hand-touched, and paintbrushed plants at the time point of stem height measurement. (B) Average stem height of plants in (A). At least 12 plants were assessed per treatment. (C) Flowering time, measured as the number of rosette leaves at the time of bolting, of plants shown in (A). (D) Brush dose response curve. At least 22 plants were assessed per treatment. This experiment was repeated once. (E) Secondary screen of T-DNA insertion lines for insensitivity to paintbrushing. Eight plants were assessed per line for each treatment. Line 6-4-2 was selected for further analysis and renamed *touch-insensitive 1 (ths1).* This experiment was not replicated but seeds were collected to assess in the next generation. (F) *ths1* mutants are heritably insensitive to touch. Twenty plants were assessed per genotype for each treatment. Error bars indicate standard deviation. This experiment was repeated twice with similar results. In (B) and (D), asterisks mark difference from untouched controls, *P*<0.01 (Student’s *t*-test). In (E) and (F), statistical groups represented with letters were determined by ANOVA followed by Tukey’s HSD test, *P*<0.05.

**Fig. 2.**
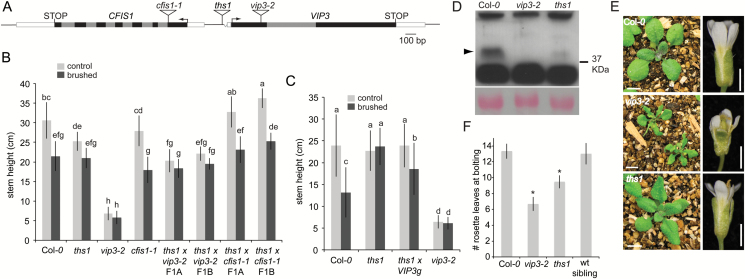
**Touch insensitivity in the *ths1* mutant is due to partial loss of function of the *VIP3* gene.** (A) Schematic of a region of chromosome 4 containing *VIP3* and *CFIS1* in *ths1*, *vip3-2* (SALK_083364) and *cfis1-1* (SAIL_367_F03) mutants. Black and grey boxes indicate exons and introns, respectively. Note that the deletion in the 5’UTR of *VIP3* is present only in the *ths1* allele. Arrows indicate transcriptional start sites. White boxes are 5’ and 3’ UTRs. (B) Complementation test, comparing stem height in parental and F1 lines in response to 8 days of paintbrushing as described in the legend to Fig. 1. At least 15 plants were used per treatment for each genotype. Error bars indicate standard deviation. This experiment was repeated once with similar results. Statistical groups represented with letters were determined by ANOVA followed by Scheffé’s test, *P*<0.05. (C) Partial rescue of touch insensitivity in the *ths1* background with *VIP3g. n*=50 plants per treatment for each genotype. Error bars indicate standard deviation. Statistical groups represented with letters were determined by ANOVA followed by Scheffé’s test, *P*<0.05. This experiment was repeated twice with similar results. (D) Top, anti-VIP3 immunoblot. Arrow indicates the band corresponding to VIP3. Bottom, Ponceau S staining of the large subunit of Rubisco. (E) Three-week-old seedlings (left) and flowers (right) from the indicated genotypes grown in soil under 24 hours of light. (F) Number of rosette leaves prior to bolting in the indicated genotypes grown in 16 hours of light. *n*=30 plants per genotype. Asterisks mark significant difference from the untouched control *P*<0.01 (Student’s *t*-test). Error bars indicate standard deviation.

**Fig. 3.**
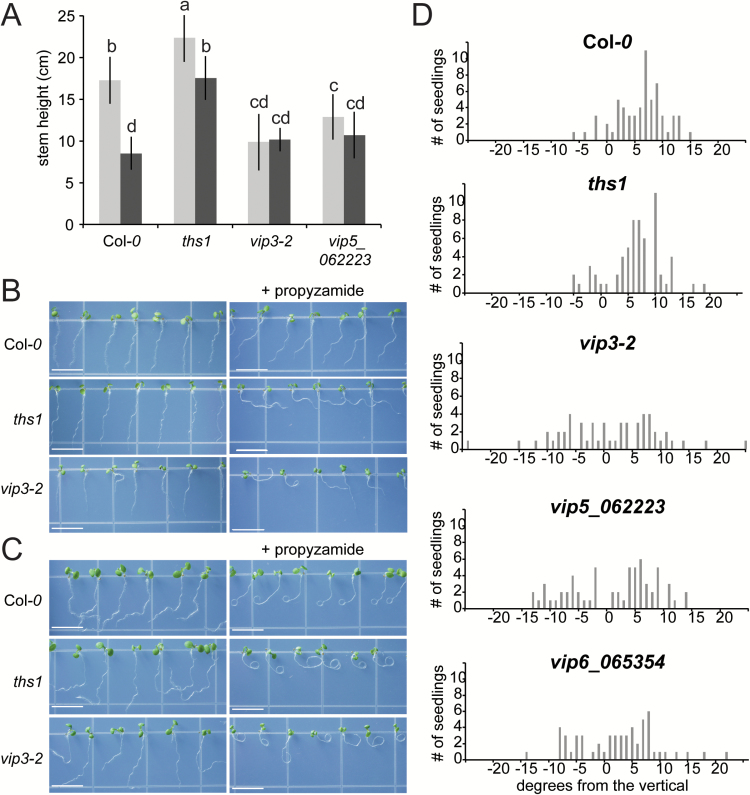
**The Paf1 complex is required for stem and root thigmomorphogenesis.** (A) Comparison of Col*-0*, *ths1*, *vip3-2* and *vip5_062223* plants after no touch (light bars) and after 8 days of paintbrushing (dark bars). Plants were grown under long day conditions of 16 hours of light in order to produce taller plants. At least seven plants were used for each genotype and treatment. Statistical groups represented with letters were determined by ANOVA followed by Scheffé’s test, *P*<0.05. Error bars indicate standard deviation. This experiment was also conducted in 24 hours of light with similar results. (B) In the slanting assay, wild type and mutant seedlings were grown horizontally on normal media or media supplemented with 3 μM propyzamide for 2 days, then tilted 30 degrees back from the vertical for 3 days. Size bar, 7 mm. (C) In the coiling assay, wild type and mutant seedlings were grown horizontally on normal media or media supplemented with 3 μM propyzamide for 2 days, then placed vertically for 3 days. (D) Distribution of root slanting angle in Col*-0* and *vip* mutant seedlings in the absence of propyzamide. Data from three independent experiments are included in each chart, providing a total of *n*=55 seedlings for each genotype.

**Fig. 4.**
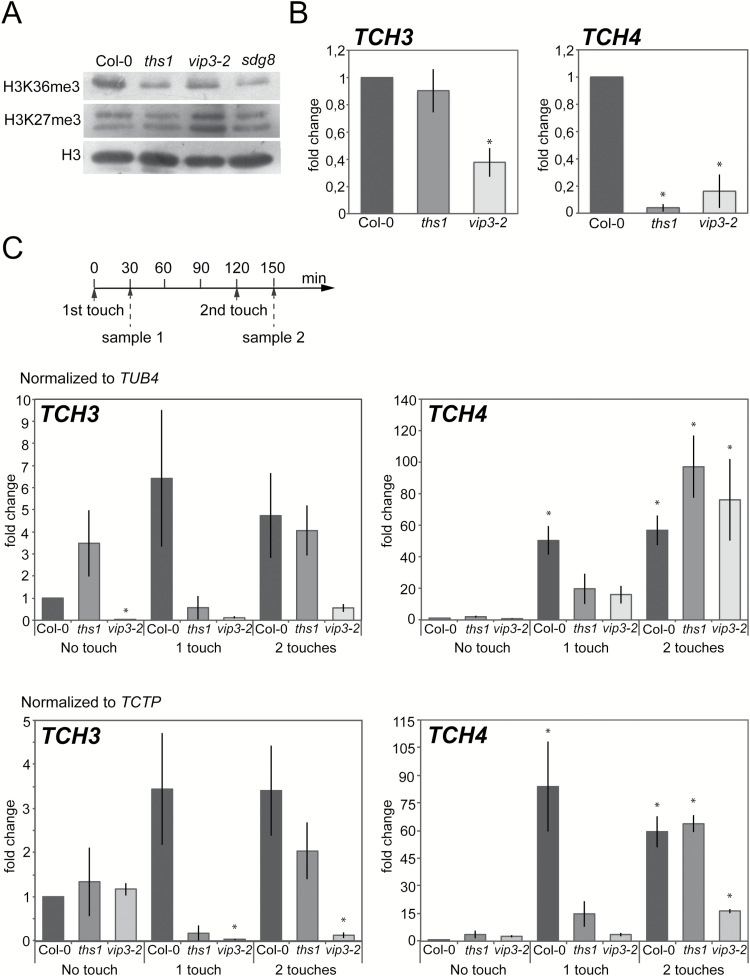
***ths1*** and ***vip3-2*** mutants have altered global and genic histone H3 methylation patterns and fail to induce *TCH3* and *TCH4* in response to touch. (A) Chromatin extracts from cauline leaves of the indicated genotypes were separated by SDS-PAGE and detected with antibodies specific to H3K36me3 or H3K27me3. (B) Relative enrichment for H3K36me3 at the *TCH3* and *TCH4* loci in cauline leaf chromatin. Chromatin was isolated as in (A), then immunoprecipitated with the H3K36me3 antibody or without antibody and precipitated DNA amplified by qRT-PCR. Data presented is a percentage of the input, normalized to the *S-ADENOSYLMETHIONINE SYNTHASE* (*SAM*) gene and represented as a fold difference with the wild type non-treated sample. Error bars indicate the SEM for three biological replicates with two technical replicates each. (C) Quantitative reverse-transcriptase PCR analysis of *TCH3* and *TCH4* expression in response to touch in wild type and mutant seedlings. Seedlings were brushed for 2 minutes with a paintbrush according to the scheme shown in the top panel. RNA was prepared from the aerial tissues of treated and untreated plants and cDNA amplified with gene specific primers. The data was normalized to *TUB4* (upper panels) or *TCTP* (lower panels) as reference genes and is presented as fold change compared to wild type non-treated samples. The error bars indicate SEM from three replicates. Asterisks mark significant difference from the untouched control *P*<0 05.

For Fig. 1 and Fig. 2, plants were grown for one week in long day conditions of 16 hours of light and 8 hours of darkness at 23°C, then transferred to 24 hours of light at 25°C for one more week. These conditions were established during the screen and were replicated in later experiments to reduce variability due to growth conditions. Seedlings were allowed to germinate and grow in a large 16-hour chamber, then moved to a smaller 24-hour chamber that was set aside only for touch assay experiments. Two-week-old seedlings were then subjected to touch stimulus for 8–10 days, stopping when bolts were visible. For hand touching, a gloved hand was held parallel to the tray and moved across the pots, touching the tops of all plants within the tray with the palm once and then repeated for a total of ten passes each day. For paintbrushing, seedling leaves were brushed each day with ten passes of a 3-inch standard paintbrush held at a 45 degree angle to the soil surface. Touched and untouched plants of the same genotype were grown side-by-side in the same tray and trays were rotated 180 degrees every day to reduce variability in temperature and airflow within the growth chambers. Stem height was measured 10–14 days later, whenever the stem height of wild type untouched plants reached over 15 cm high or approximately 30 days after germination. The number of days required for stems to reach 15 cm was dependent on the light and temperature conditions of the particular experiment and chamber used; similar results were obtained when wild type untouched stems were measured anytime beyond this point. In Fig. 3, plants were grown in 16 hours of light in order to allow the null *vernalization independence* (*vip*) mutants to grow as tall as possible. In Fig. 4, 12-day-old seedlings were grown in long day conditions of 16 hours light and 8 hours darkness at 21°C and were either untouched or touched with a large, soft paintbrush every 5–7 seconds for 2 minutes, with on average 18 brushes per treatment. Material was collected 30 minutes after the first touch treatment or 30 minutes after plants were subjected to a second touch treatment 2 hours following the first one.

### Identifying T-DNA

The left border of the T-DNA in *ths1* plants was amplified with left border primers and AD1 by TAIL PCR as described (Liu *et al.*, 1995). The third round TAIL PCR product was cloned using the pGEM-T Easy vector kit (Promega) and sequenced. The location of the T-DNA was verified by PCR genotyping. Oligos 29830.F and 29830.R were used to amplify the left border region from genomic DNA for sequencing and independent verification of the junction.

### Characterizing ths1 T-DNA

Templates in PCR reactions were either 1 μl genomic DNA or 1 μl cDNA made from 5 mg leaf tissue from wild type or *ths1* mutant plants. PCR reactions were carried out using Hot Star Taq (Qiagen) and the following primer pairs: 29830-QPCR.F2/ 29830.F3, 29830-QPCR.F2/LBb1, 29830-QPCR.F2/Lba1, or ACTF2/ACTR2. The 29830-QPCR.F2/29830.F3 product includes an intron of approximately 450 bp from the *VIP3* gene and the ACTF2/ACTR2 product includes an intron approximately 100 bp from the *ACT* genes. 5–10 μl of each PCR reaction was separated on an ethidium gel and photographed.

### Mutant lines

The *cfis1*-1 (SAIL_367_F03), *vip3*-2 (SALK_083364), *vip5-062223* (SALK_062223) and *vip6-065364/elf8-2* (SALK_065364) lines were obtained from the Arabidopsis Biological Resource Center. For genotyping, plant genomic DNA was isolated as described (Edwards *et al.*, 1991) and wild type or mutant alleles amplified using the primer combinations listed in Supplementary Table S1 at *JXB* online.

### VIP3 immunoblotting

50 mg of leaf tissue from 3–4 week old wild type, *vip3-2*, or *ths1* mutant plants was ground in liquid nitrogen and immediately resuspended in 200 μl 2X SDS PAGE sample buffer. Equal volumes were run on an 8% SDS PAGE gel, blotted to polyvinylidene difluoride (PVDF), blocked in 5% milk/Tris-buffered saline with Tween 20 (TBST), and probed with a 1:1000 dilution of anti-VIP3 antibody overnight at 4°C. The blot was washed three times for 30 minutes each with TBST and incubated with anti-Rabbit HRP (Sigma) at 1:5000 for 60 minutes at room temperature, then washed again with TBST for 30 minutes. Peroxidase activity was detected with the SuperSignal West Femto kit (Thermo Scientific) and imaged on X-ray film. Equal loading was confirmed by Ponceau S staining and by comparison of non-specific bands.

### Root slanting and coiling

Seeds were sown on square 100 x 15 mm plates (Falcon) containing 0.5X Murashige and Skoog medium, 1% sucrose and 1.6% phytagar, and in some cases with 3 μM propyzamide (Sigma). Plates were sealed with porous tape. After incubation at 4°C for 2 days in the dark, plates were transferred to a growth chamber and grown vertically for 2 days and then tilted 30° back from the vertical for 2–3 days. All plates were grown in 16 hours of light at 23°C. For curling assays, seedlings were grown on the same media for 2 days vertically and then 3 days horizontally.

### Chromatin immunoprecipitation

All experiments were performed in triplicate with two technical repeats for each of the three biological repeats. For ChIP, 0.5 g of cauline leaf material was harvested from plants grown for three weeks under short day conditions of 8 hours light and 16 hours dark at 21°C followed by 2 weeks under continuous light at 16°C (as shown previously (Zhang *et al.*, 2003). These conditions attenuate the phenotypic defects of the *vip3* mutant, allowing us to collect sufficient tissue. Harvested tissue was placed on ice and crosslinked in 40 ml of infiltration buffer (13.69 g sucrose, 1 ml 100 mM PMSF, 1 ml 1M Tris/HCl pH 8, 200 μl 0.5M EDTA, 2.7 ml 37% formaldehyde in 100 ml distilled water). Vacuum was slowly introduced to the samples on ice, maintained for 10 minutes and slowly released. This was repeated three times. To stop the reaction 2 ml of 2M glycine was added to each sample. The samples were then infiltrated again for 5 minutes. Samples were washed with 500 ml of distilled water, dried on filter paper and frozen in liquid nitrogen.

Extraction of nuclei was performed as described (Jaskiewicz *et al.*, 2011) in extraction buffer containing 0.44 M sucrose, 1.25% Ficoll, 2.5% Dextran T40, 20 mM Hepes KOH pH 7.4, 10 mM MgCl_2_, 0.5% Triton X-100, 5 mM DTT, 1 mM PMSF and 1X complete protease inhibitor cocktail (Roche, Product No 04693116001). After spinning down, the pellet was diluted in 500 µl of lysis buffer (50 mM Tris-HCl pH 8, 10 mM EDTA, 1% SDS and 1X complete protease inhibitor cocktail) and sonicated with a Bioruptor UCD-200 to fragments of 0.2–0.5 kilobases.

8 µg of chromatin diluted to a total volume of 500 µl with dilution buffer [1.1% Triton X-100, 1.2 mM EDTA, 16.7 mM Tris-HCl pH 8, 167 mM NaCl, 0.2% complete protease inhibitor cocktail (Roche, EDTA-Free, Product No 04693116001)] was used for immunoprecipitation with specific antibodies on Protein G Plus-Agarose beads (Santa Cruz Technology, sc2002L) overnight on a spinning wheel at 4°C. Two independent immunoprecipitations with the H3K36me3 antibody (Abcam: ab9050, 3 µl per sample) and a no-antibody control were performed using each chromatin sample. Three independent chromatin samples were prepared for each genotype. Following the incubation, all samples were washed with wash buffer (150 mM NaCl, 20 mM Tris HCl pH 8, 2 mM EDTA, 1% Triton X-100, 0.1% SDS, 1 mM PMSF and 1X complete protease inhibitor cocktail). Precipitated DNA was extracted with the NucleoSpin PCR purification kit (Machery-Nagel REF-740809.250).

The quantity of DNA in each sample was determined using quantitative reverse-transcriptase PCR with the LightCycler 480 SYBR Green I Master Mix (Roche, Product No 04887352001) and the primers listed in Supplementary Table S1 to amplify a portion of exon 2 of *TCH3*, or the junction between exon 1 and exon 2 of *TCH4*. Enrichment for H3K36me3 at each region of interest was initially calculated as a percentage of the DNA recovered in the corresponding input samples. All samples were normalized against the enrichment for H3K36me3 at the *S-ADENOSYL METHIONINE SYNTHASE* (*SAM*; At4g01850) locus (Cazzonelli *et al.*, 2014). The signal intensity after immunoprecipitation with specific antibodies was at least 10 times higher than with the no-antibody control for the corresponding samples. The final result is represented as a fold difference over the wild type sample.

### Chromatin immunoblotting

2.5 µg of chromatin extract from 0.5 g of cauline leaf tissue, prepared as described above, from each sample were run on a 10% SDS PAGE gel, blotted to PVDF, blocked in 3% BSA/phosphate-buffered saline (PBS) overnight at 4°C and probed with a 1:5000 dilution of each primary antibody. The antibodies Abcam ab9050 and mAbcam ab6002 were used to detect H3K36me3 and H3K27me3, respectively. Anti-Histone H3 antibody (Agrisera, AS10710) was applied as a loading control. After washing the membranes with 0.5% BSA, 0.1% Tween-20 in PBS, they were probed with a secondary anti-Rabbit HRP (Sigma) antibody at 1:10 000 for 90 minutes at room temperature. The signal was visualized with the Pierce™ ECL Western Blotting Substrate (ThermoFisher Scientific, No32106)

### Quantitative RT-PCR (qPCR)

All experiments were performed in triplicate, with three technical repeats for each of the three biological repeats. For Fig. 4, RNA extraction from the aerial portion of 12-day-old plants grown under long day conditions of 16 hours of light and 8 hours of dark at 21°C was performed with the Spectrum^™^ Plant Total RNA Kit (STRN250 SIGMA). 800 ng of total RNA was used for cDNA synthesis with the oligo(dT)_20_ primer and RevertAid Reverse Transcriptase (Thermo Scientific #EPO441). Expression of target genes was measured using LightCycler 480 SYBR Green I Master Mix (Roche, Product No 04887352001) in a StepOne Cycler (Applied Biosciences) in a reaction volume of 15 µl. The sequences of primers designed to amplify the coding regions of *TUB4* (AT5G44340), *TCTP* (AT3G16640)*, TCH3* (AT2G41100), and *TCH4* (AT5G57560) are listed in Supplementary Table S1. *TUB4* and *TCTP* are classically used as reference genes for normalization because of their relatively invariant expression (Lee *et al.*, 2005; Szecsi *et al.*, 2006). Data were analyzed by the ΔΔC_T_ approach with *TCTP* or *TUB4* as reference genes.

For Supplementary Fig. S1, cDNAs were generated using an oligo(dT)_20_ primer and M-MLV Reverse Transcriptase (Promega) from template RNA extracted from rosette leaves of 4-week-old plants with TRIzol Reagent (Invitrogen). Primer mixes designed to amplify *CFIS1* (29820-QPCR.F/29820-QPCR.R), exon 1 of *VIP3* (29830-QPCR.F/29830-QPCR.R), exon 2 of *VIP3* (29830-QPCR.F3/29830-QPCR.R3), or *ACTIN2/7/8* (ACTF-QPCR/equal volumes of Actin2.R-QPCR, Actin7.R-QPCR and Actin8.R-QPCR) were added to a cocktail containing 1X SYBR Green PCR Master Mix (Applied Biosciences) and 0.5 μl cDNA to make a final 25 μl reaction. After amplification, the data were analyzed using StepOne software (Applied Biosciences).

### Statistical analyses

ANOVAs and regressions were performed in R with the agricolae and car packages. Type II sum of squares was used for two-way ANOVAs and Tukey’s or Scheffé’s methods used as post-hoc means separation tests for balanced and unbalanced datasets, respectively. Student’s t-tests were performed in Excel.

## Results

### A forward genetic screen identifies a mutant defective in stem thigmomorphogenesis

The leaves of two-week-old soil-grown *Arabidopsis thaliana* seedlings, ecotype *Columbia*-*0* (*Col-0*), were mechanically stimulated either by touching or brushing with a paintbrush. Stimulus was only administered to rosette leaves prior to bolting. We found that paintbrushing was almost as effective as hand touching at reducing subsequent stem height (Fig. 1A and 1B) and had the advantage of avoiding contact between experimenter and plant. The difference in stem heights between touched or paintbrushed and control plants was maintained until senescence. Repeated touch can lead to a delay in flowering (Chehab *et al.*, 2012; Lange and Lange, 2015) but in our hands flowering time was not significantly different between hand-touched or paintbrushed plants and untouched plants (Fig. 1C). The reason for this discrepancy is not clear, though it is possible that it can be attributed to differences in growth conditions or in the severity of the mechanical stimulus. As expected, the decrease in stem height was proportional to the number of days of stimulus (Fig. 1D).

Twenty-four pools of 100 *pROK2* T-DNA insertion lines (Alonso *et al.*, 2003) were sown in flats and all seedlings subjected to 10 days of paintbrushing prior to bolting as described above. Thirty-three plants with stems taller than the wild type after touch were selected for further analysis. A representative example of a secondary screen in the next generation is shown in Fig. 1E. Line 6-4-2 showed insensitivity to touch as well as stem height similar to Col-*0* and was named *touch insensitive 1 (ths1).* Twenty *ths1* siblings were tested in the next generation and heritable insensitivity to touch was confirmed (Fig. 1F).

### Touch insensitivity is caused by a T-DNA insertion located in the 5’UTR of the *VERNALIZATION INDEPENDENCE 3 (VIP3*) gene.

Using thermal asymmetric interlaced PCR (Liu *et al.*, 1995), we located a T-DNA insertion in the *ths1* genome on chromosome 4, in the presumptive promoter of *VERNALIZATION INDEPENDENCE 3 (VIP3*) (At4g29830) (Fig. 2A). The first 23 bp of the *VIP3* 5’UTR were deleted, placing the left border of the T-DNA 57 bp upstream of the ATG of *VIP3* and 332 bp upstream of the ATG of At4g29820 (*CFIS1*), which is expressed from the other strand of chromosome 4. We were unable to amplify the right border of the T-DNA. This T-DNA insertion upstream of *VIP3* segregated with the observed touch insensitivity in *ths1* mutants. When *ths1* mutant plants were backcrossed to wild type Col*-0* plants and the F2 progeny screened for inheritance of the T-DNA, the seven F2 lines homozygous for the T-DNA did not significantly respond to touch by reducing stem height, while F2 lines lacking the T-DNA did (see Supplementary Fig. S2). *VIP3* encodes a WD-repeat protein that is implicated in chromatin remodeling, mRNA turnover, flowering time and vernalization (Zhang *et al.*, 2003; Oh *et al.*, 2004; Dorcey *et al.*, 2012; Takagi and Ueguchi, 2012). *CFIS1* encodes a protein proposed to function as a cleavage factor during mRNA polyadenylation (Hunt *et al.*, 2008).

To determine which gene was responsible for the touch insensitive phenotype of the *ths1* mutant, homozygous *vip3*-2 (Jolivet *et al.*, 2006) and *cfis1-1* (Fig. 2A and Supplementary Fig. S1) mutants were crossed to homozygous *ths1* mutants and the F1 generation analyzed for stem touch responsiveness as in Fig. 1F. The offspring of two independent crosses between *cfis1-1* and *ths1* mutants were touch sensitive, ruling out *CFIS1* as responsible for touch sensitivity in the simplest genetic scenario and establishing that *ths1* is a recessive mutation (Fig. 2B). However, the offspring of two independent crosses between *vip3-2* and *ths1* mutants were touch insensitive, implicating *VIP3* as responsible for touch insensitivity in *ths1* mutants (Fig. 2B). The introduction of a transgene encoding a wild type copy of the *VIP3* gene partially rescued touch sensitivity in the *ths1* mutant background (Fig. 2C). Taken together, these data demonstrate that a defect in the *VIP3* locus is responsible for the touch insensitive phenotype of the *ths1* mutant. We therefore renamed *ths1* as *vip3-6*, with other existing alleles named *vip3-1, vip3-2, vip3-3, vip3*^*zwg*^*and bouquet-1* (Zhang *et al.*, 2003; Jolivet *et al.*, 2006; Dorcey *et al.*, 2012; Takagi and Ueguchi, 2012).

Immunoblotting with an anti-VIP3 antibody (Zhang *et al.*, 2003) was performed on whole cell extracts from the leaves of wild type, *ths1* and *vip3-2* plants. A VIP3-specific band of approximately 34 kDa present in extracts from wild type plants and absent from extracts from *vip3-2* plants was only faintly detectable after long exposures in extracts from *ths1* plants (Fig. 2D). We used quantitative RT-PCR to compare transcript levels from the first or second exon of *VIP3* and exons 1–3 of *CF1S1* in *ths1*, *vip3-2*, and wild type plants. The *ths1* mutant showed a modest 1.5 to 3-fold increase in *VIP3* and *CFIS1* transcript levels compared to wild type (see Supplementary Fig. S3A). We note that the data shown in Fig. 2B show that the touch insensitive phenotype of *ths1* is not due to a recessive defect in *CFIS1* and that the *ths1* phenotype is not dominant; thus this modest overexpression of *CFIS1* due to the T-DNA insertion is unlikely to be responsible for the observed phenotype.

Semi-quantitative RT-PCR detected an abundant transcript stretching from the left border of the T-DNA to the second exon of *VIP3*, along with several larger, less abundant transcripts (see Supplementary Fig. S3B). These transcripts may be derived from the NOS or CaMV 35S promoters present inside the pROK2 vector. Though present at levels similar to or higher than the wild type transcript, this transcript may lack proper start site or splicing information due to its aberrant start site. Thus, while *ths1* plants produce mRNA from the *VIP3* locus, it is unstable and/or not properly translated.

*vip3-2* mutants are small with narrow, crinkled leaves and short, misshapen floral organs, as shown previously (Jolivet *et al.*, 2006). *ths1* mutants grown side-by-side were smaller and had narrower leaves than wild type plants and exhibited mild floral organ defects (Fig. 2E). We also assessed the flowering time of wild type and *ths1* homozygous mutant siblings derived from a *ths1* x Col*-0* backcross, Col*-0*, and *vip3-2* mutants. *ths1* mutant siblings showed slightly but significantly earlier flowering, while wild type siblings were indistinguishable from Col*-0*, and *vip3-2* mutants had a clear early flowering phenotype (Fig. 2F). Taken together, the data presented in Fig. 2 show that an intermediate loss-of-function allele of *VIP3* is responsible for touch insensitivity in *ths1* mutants. From these results we infer that the low amount of VIP3 protein produced in the *ths1* background (Fig. 2D) is sufficient for most developmental functions of VIP3.

### Two members of the Paf1 complex are required for stem thigmomorphogenesis

The VIP3 protein has been detected both in the Paf1 complex and in the Superkiller (SKI) complex (Dorcey *et al.*, 2012). Several other proteins (VIP4, VIP5, VIP6/ELF8 and VIP2/ELF7) are known to be part of the Paf1 complex but not the SKI complex (Zhang and van Nocker, 2002; Zhang *et al.*, 2003; He *et al.*, 2004; Oh *et al.*, 2004; Oh *et al.*, 2008; Shiraya *et al.*, 2008; Park *et al.*, 2010). To determine if the touch insensitivity of *ths1* can be attributed to the action of the Paf1 complex, we tested null *vip5-062223* mutants (Oh *et al.*, 2004) in the paintbrush assay. When grown in 16 hours of light to produce taller plants, *vip3-2* and *vip5-062223* mutants were similarly touch insensitive, while wild type plants still showed an approximately 40% reduction of stem height when touched (Fig. 3A). Early flowering is not sufficient to confer touch insensitivity as two early flowering lines showed a normal touch response (Supplementary Fig. S4).

### Root skewing and coiling is abnormal in *ths1* and *vip* mutants

Assays for root touch response that involve growing seedlings on agar plates include waving (Okada and Shimura, 1990), skewing or slanting (Simmons *et al.*, 1995; Rutherford and Masson, 1996), and curling or looping (Mirza, 1987; Simmons *et al.*, 1995; Buer *et al.*, 2000). These assays are thought to reveal the interplay between the response to touching of the agar surface and the response to gravity (Okada and Shimura, 1990; Thompson and Holbrook, 2004). Forward and reverse genetic approaches have revealed that slanting is affected by a diverse array of pathways, including the cortical microtubule array, hormones such as auxin and ethylene and environmental factors such as sucrose and salt stress (Buer *et al.*, 2000; Shoji *et al.*, 2006 ; Oliva and Dunand, 2007). In addition, lateral root initiation can be modulated by physical bending with forceps or by growth into barriers (Ditengou *et al.*, 2008; Laskowski *et al.*, 2008; Richter *et al.*, 2009). This touch perception pathway appears to modulate the endogenous auxin-regulated pathway for lateral root initiation (Ditengou *et al.*, 2008; Laskowski *et al.*, 2008; Richter *et al.*, 2009).

According to publically available gene expression databases, components of the Paf1 complex are widely expressed in most plant tissues, including the root (Winter *et al.*, 2007). We therefore analyzed root thigmomorphogenetic responses using root slanting and coiling assays (Bidzinski *et al.*, 2014; Swanson *et al.*, 2015). As previously observed, the roots of wild type plants slant to the left when grown on hard agar plates tilted 30 degrees from the vertical, and this phenomenon was enhanced in the presence of the microtubule-destabilizing drug propyzamide (Furutani *et al.*, 2000; Nakamura *et al.*, 2004), while *ths1* and *vip3*-2 mutant roots slanted strongly to the right under these conditions (Fig. 3B). Similar results were obtained for root coiling assays, where seedlings were grown horizontally on hard agar plates (Fig. 3C). Plotting the distribution of slanting angle within a population of seedlings grown in the absence of propyzamide revealed that wild type plants slanted on average 6 degrees to the left when viewed from above the agar surface (Fig. 3D). While the *ths1* mutant did not show a clear difference from wild type, *vip3-2*, *vip5-062223* and null *vip6-065364 (elf8-2*) (He *et al.*, 2004; Oh *et al.*, 2004; Shiraya *et al.*, 2008) mutants showed a dramatic difference in how their roots responded to growth on hard agar, with a wider range of slanting angles and no preference for slanting direction. In summary, *ths1* mutants have defects in root skewing and coiling assays when propyzamide is added, and *vip* null mutants have defects in root skewing and coiling assays both with and without propyzamide.

### *ths1 and vip3-2* mutants exhibit a histone H3 trimethylation pattern associated with inactive chromatin globally and at *TCH3* and *TCH4* loci

The Paf1 complex is known to promote histone methylation patterns associated with active chromatin. At *Flowering Locus C* (*FLC*), the Paf1 complex promotes lysine methylation of histone H3 associated with transcriptionally active regions, lysine 4 (H3K4) and lysine 36 (H3K36), while reducing lysine methylation associated with transcriptional repression at lysine 27 (H3K27) (Zhao *et al.*, 2005; Ko *et al.*, 2010). Global levels of trimethylation at H3K36 and H3K27 in cauline leaves were assessed by western blotting with specific antibodies. Under these conditions, a reduction in the level of H3K36 trimethylation (H3K36me3) was detected in *ths1* and *vip3-2* mutants (Fig. 4A). As a positive control, we also observed reduced H3K36me3 levels in the *sdg8* background under the same conditions (Fig. 4A); *SDG8* encodes an H3K36-directed methyltransferase and is required for global and genic H3K36me3 (Zhao *et al.*, 2005; Xu *et al.*, 2008). H3K27me3 levels were higher than the wild type in *vip3* mutants but indistinguishable from the wild type in *ths1* and *sdg8* mutants.

H3K36me3 levels were also assessed by chromatin immunoprecipitation at *TCH3* and *TCH4* loci. *TCH3* is one of the most studied *TCH* genes and encodes a calmodulin-like calcium-binding protein (Sistrunk *et al.*, 1994). *TCH4* encodes a xyloglucan endotransglycosylase (Xu *et al.*, 1995). We found that levels of H3K36me3 were not significantly changed at the *TCH3* locus in *vip3-6/ths1*. However, in the null *vip3-2* mutant they were reduced two-fold compared to the wild type (Fig. 4B). Levels of H3K36me3 were significantly reduced at the *TCH4* locus in both the *vip3-6/ths1* and *vip3-2* mutants when compared to wild type.

To determine if these chromatin marks were related to *TCH* gene expression, we tested the rapid touch induction of *TCH4*. Thirty minutes after one touch treatment with a paintbrush, a significant induction of *TCH4* expression was observed in the wild type (Fig. 4C). The same trend, albeit not statistically significant, was observed for *TCH3*. Two touch treatments also led to high levels of expression of both *TCH* genes in the wild type (Fig. 4C). These responses were reduced and/or delayed in both *vip3* mutants (Fig. 4C). Note that the induction was not totally absent in both *vip3* mutants and could even reach wild type levels after two subsequent touches for *TCH4* expression in the *vip3-6/ths1* mutant. Nonetheless, the induction was never detected after a single touch event in the *vip3* mutants (Fig. 4C). *VIP3* transcript levels were not significantly altered in response to touch (Supplementary Fig. S5). Thus, the Paf1 complex is not only required for long-term thigmomorphogenesis responses but is also involved in short-term touch-induced transcription.

## Discussion

We show here that the Arabidopsis Paf1 complex is a critical factor for stem and root thigmomorphogenesis, stereotypical alterations in plant form that are made in response to repeated mechanical disturbance. We also show that THS1/VIP3 is required for both global and genic levels of H3K36 methylation and for upregulation of two canonical touch-inducible loci in response to touch stimulation. *VIP3* was identified in a genetic screen with no *a priori* expectations and our findings are consistent with previous observations that *sgd8* mutants exhibit defects in wind-induced alterations in leaf morphology and in H3K4 trimethylation at the *TCH3* gene (Cazzonelli *et al.*, 2014). It has been proposed that mitotically stable chromatin marks serve to establish and stabilize certain transcriptional states (Iwasaki and Paszkowski, 2014; Alabert *et al.*, 2015; Avramova, 2015; Crisp *et al.*, 2016). The data presented here are consistent with this model, demonstrating that the state of chromatin is modulated by the Paf1 complex and facilitates both the spatial and temporal aspects of thigmomorphogenesis, namely its systemic nature and need for repetitive touch.

### Linking VIP3 and the Paf1 complex to stem and root thigmorphogenesis.

We present four lines of evidence to support the conclusion that a lesion in *VIP3* is responsible for the thigmomorphic defects seen in *ths1* mutants: 1) The T-DNA inserted into the *VIP3* 3’ UTR in *ths1* mutants segregates with its touch insensitive phenotype, ruling out a causal mutation at a second site (Supplementary Fig. S2); 2) While the T-DNA insertion is associated with a modest increase in expression of both *VIP3* and *CFIS1* (Supplementary Fig. S3A), the complementation test shown in Fig. 2B, where the *cfis1* mutant complements the *ths1* mutant but a *vip3* null mutant does not, indicates that it is a recessive lesion in *VIP3* that is responsible for the touch insensitivity of *ths1* mutants; 3) A *VIP3g* genomic construct partially restores touch responsiveness to the *ths1* mutant. This result is difficult to explain if a gene other than *VIP3* were involved given that no *CFIS1* sequences were included in the *VIP3g* construct; and 4) *vip3-2* null mutants do not respond to touch.

These lines of evidence link VIP3 to stem thigmomorphogenesis. We also present evidence that other components of the Paf1 complex are required for thigmomorphic responses in both the stem and the root. The stems of *vip3-2* and *vip5* null mutants do not shorten in response to paintbrushing (Fig. 3A) and *ths1*, *vip3-2, vip5,* and *vip6* mutants show defects in root coiling and slanting assays (Fig. 4). However, we cannot rule out the possibility that *vip* null mutants are simply too short, even when grown in 16 hours of light, to get any shorter. Furthermore, we acknowledge that the root coiling and slanting assays used here do not solely assess touch responses but also integrate the perception of gravity (Thompson and Holbrook, 2004). Nonetheless, these additional data help support our hypothesis that the plant Paf1 complex is required for normal thigmomorphogenesis in the stem and in the root.

One challenging aspect of the paintbrush assay used here is the variability in stem height; both in the average untouched *ths1* stem heights compared to average untouched wild type stem heights and in the extent to which stem height in the *ths1* mutant is insensitive to touch (compare Fig. 1F, 2B, 2C and 3A). We found that the average stem height of any genotype depends on a number of growth conditions including, but not limited to, light and temperature. We controlled for these variables as much as possible by using the same shelf and chamber, rotating trays and growing touched and untouched pots side-by-side. We were nonetheless unable to completely prevent stem height variability. However, we note that the key comparison in this study is between touched and untouched individuals of a single genotype, and that *ths1* mutants were consistently less sensitive to paintbrushing than the wild type under all conditions tested.

### The Paf1 complex as a conserved and multitasking regulator of gene expression

First identified in yeast as a transcriptional regulator, the Paf1 complex was later shown to be present and functionally conserved in all kingdoms (Koch *et al.*, 1999; Penheiter *et al.*, 2005; Sheldon *et al.*, 2005; Nordick *et al.*, 2008; Oh *et al.*, 2008; Dermody and Buratowski, 2010; Chu *et al.*, 2013; Sadeghi *et al.*, 2015). The Paf1 complex plays a role in a number of transcription-related processes, such as facilitating elongation, recruitment and regulation of chromatin-modifying factors like histone-methylation factors, transcription termination and polyadenylation (Krogan *et al.*, 2003; Ng *et al.*, 2003; Nordick *et al.*, 2008; Penheiter *et al.*, 2005; Rozenblatt-Rosen *et al.*, 2009; Zhang *et al.*, 2009; Tomson and Arndt, 2013; Sadeghi *et al.*, 2015). In yeast, it was also shown to participate in small nucleolar RNA formation (Sheldon *et al.*, 2005; Tomson *et al.*, 2013). Paf1 complex activity has been reported to be necessary for the maintenance of the active chromatin marks histone H3K4me3 and H3K36me3 in yeast, plant, human and mouse cells (Jaehning, 2010; Zhang *et al.*, 2013), providing further evidence for its functional conservation among kingdoms.

Impaired Paf1 complex function leads to developmental defects in animals. The activity of the Paf1 complex is required for heart and neural crest development in zebrafish (Nguyen *et al.*, 2010), embryonic epidermal morphogenesis in *C. elegans* (Kubota *et al.*, 2014), Notch and Wnt signaling in *Drosophila* (Bray *et al.*, 2005; Mosimann *et al.*, 2009; Bahrampour and Thor, 2016), key lineage specific factor expression in mice embryos (Zhang *et al.*, 2013), oligodendrocyte differentiation (Kim *et al.*, 2012) and cardiomyocyte specification (Langenbacher *et al.*, 2011). It also affects cancer progression in human cell lines (Dey *et al.*, 2011; Tomson and Arndt, 2013) and anti-viral immune responses (Liu *et al.*, 2011). In plants, the Paf1 complex functions in the regulation of flowering time through establishment of histone marks at the *FLC* locus (Zhao *et al.*, 2005; Oh *et al.*, 2008; Xu *et al.*, 2008).

### Chromatin, a new frontier in plant mechanotransduction?

Given that we find that *TCH* gene induction in response to touch is delayed in *vip3* mutants, the function of the Paf1 complex does not seem absolutely required for *TCH* induction, but instead may prime a quick response to mechanical perturbations through histone modifications at *TCH* loci. The role of the Paf1 complex in thigmomorphogenesis might therefore reside in providing chromatin with the ability to respond to mechanical perturbations rapidly.

This scenario echoes a number of recent reports in animal systems suggesting that mechanotransduction incorporates a chromatin component. In fact, because chromatin modifications change the shape of the nucleus, notably through chromatin condensation and decondensation states, it has been proposed that mechanical forces may in turn be the primary modifiers of chromatin (Pagliara *et al.*, 2014). Global chromatin modifications have consistently been associated with nuclear stiffness [for example (Schreiner *et al.*, 2015)] and nuclear stiffness has been shown to increase with differentiation (Hampoelz and Lecuit, 2011). Given that chromatin is associated with the nuclear envelope through lamins, its modification may result from deformations originating within the extracellular matrix that are propagated through the cytoskeleton and the LINC (linker of the nucleoskeleton and cytoskeleton) complex (Isermann and Lammerding, 2013). Applying mechanical strains to mesenchymal stem cells in culture leads to a lamin-dependent decrease in histone deacetylase activity and an increase in histone acetylation (Li *et al.*, 2011). The finding that the Paf1 complex is involved in thigmomorphogenesis in plants may thus provide an opportunity to unravel potential connections between cytoskeletal and gene expression responses to mechanical perturbation.

Our results build a foundation for future investigations into the possible function of the Paf1 complex and chromatin marks in priming *TCH* genes for rapid induction upon mechanical stimulation and in translating discrete touch events into a continuous growth response. Given that the Paf1 complex is conserved across kingdoms and because animals also alter their body plan in response to repeated mechanical stresses (Kahn *et al.*, 2009; Heckel *et al.*, 2015; Steed *et al.*, 2016), these findings also raise the possibility of a common mechanism for recording multiple mechanical deformations and translating them into developmental changes.

## Supplementary Material

Supplementary Data

## Abbreviations:

*Col*-*0*: *Columbia*-*0*;
FLC: *Flowering Locus C;*
JA: jasmonic acid;
LINC: linker of the nucleoskeleton and cytoskeleton;
me3: trimethylation;
Paf1: RNA polymerase II-associated factor 1;
PBS: phosphate-buffered saline;
PVDF: polyvinylidene difluoride;
qRT-PCR: quantitative reverse-transcriptase PCR;
SAM: *S-ADENOSYLMETHIONINE SYNTHASE;*
TBST: Tris-buffered saline with Tween 20;
TCH: *TOUCH*
ths1: *touch insensitive 1;*
VIP3: *VERNALIZATION INDEPENDENCE 3*
