# Supplementary material for: The RNA Polymerase-Associated Factor 1 Complex Is Required for Plant Touch Responses
Source: J Exp Bot. 2016 Dec 15;68(3):499–511. doi: 10.1093/jxb/erw439 (PMC5441907; doi:10.1093/jxb/erw439)
Supplement: Supplementary Data [file erw439_Supplementary_Data.zip › Supplementary_Figures_S1_S5_Table_S1.pdf]

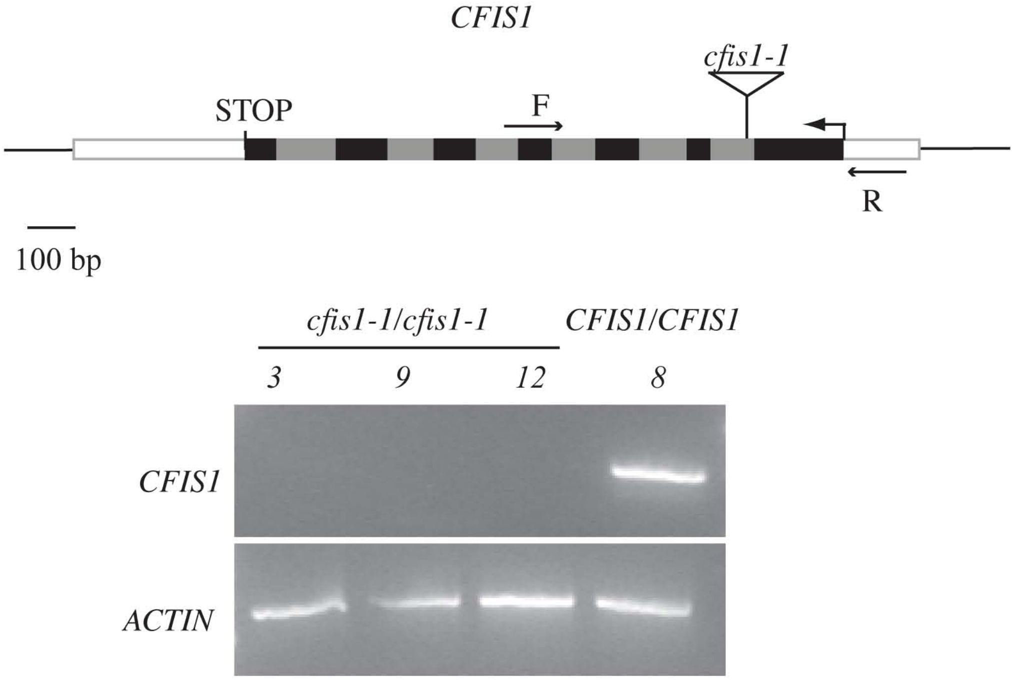

Supplementary Fig. S1. The *cfis1-1* allele produces little to no *CFIS1* transcript. Top panel, schematic of the *CFIS1* genomic sequence and the location of the *cfis1-1* T-DNA insertion allele. The *cfis1-1* allele is a T-DNA insertion predicted to be located 16 bp past the start site. Black boxes are introns, grey boxes are exons. Bottom panel, three siblings were identified as homozygous *cfis1-1* and one sibling identified as homozygous wild type *CFIS1* by PCR genotyping. cDNA prepared from leaf tissue from each of these lines was used as a template in PCR reactions amplifying either the first four exons of *CFIS1* or amplifying *ACTIN* used as a loading control.

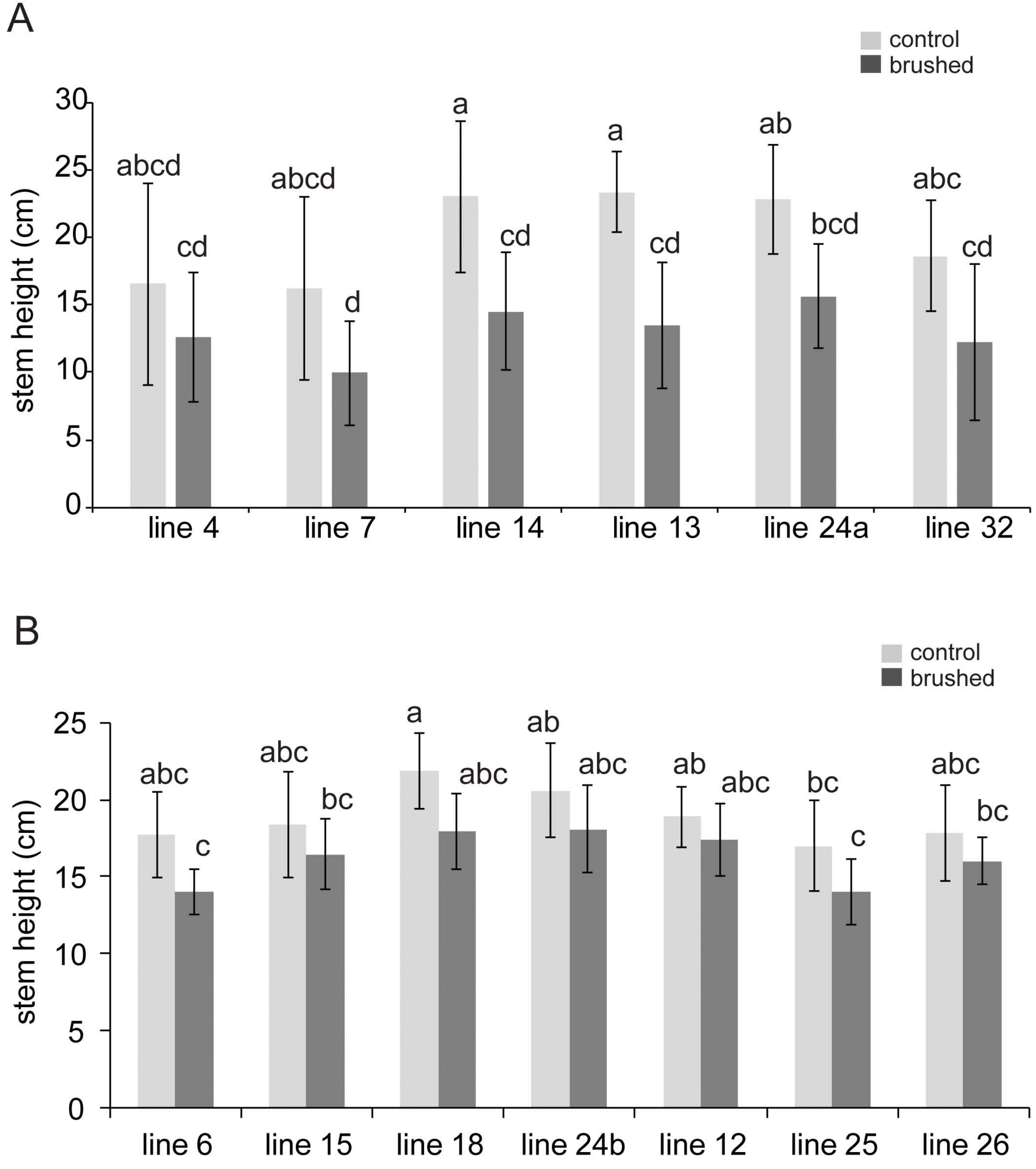

Supplementary Fig S2. T-DNA in the *VIP3* gene is linked to the touch insensitive phenotype. Comparison of F2 plants wild type at the *VIP3* locus (A) and F2 plants homozygous for the T-DNA insertion in the *VIP3* gene (B), after no touch (light bars) and after 10 days of brushing (dark bars). At least 15 plants were used for each genotype and treatment. Statistical groups represented with letters were determined by ANOVA followed by Scheffé's test, $P<0.05$.

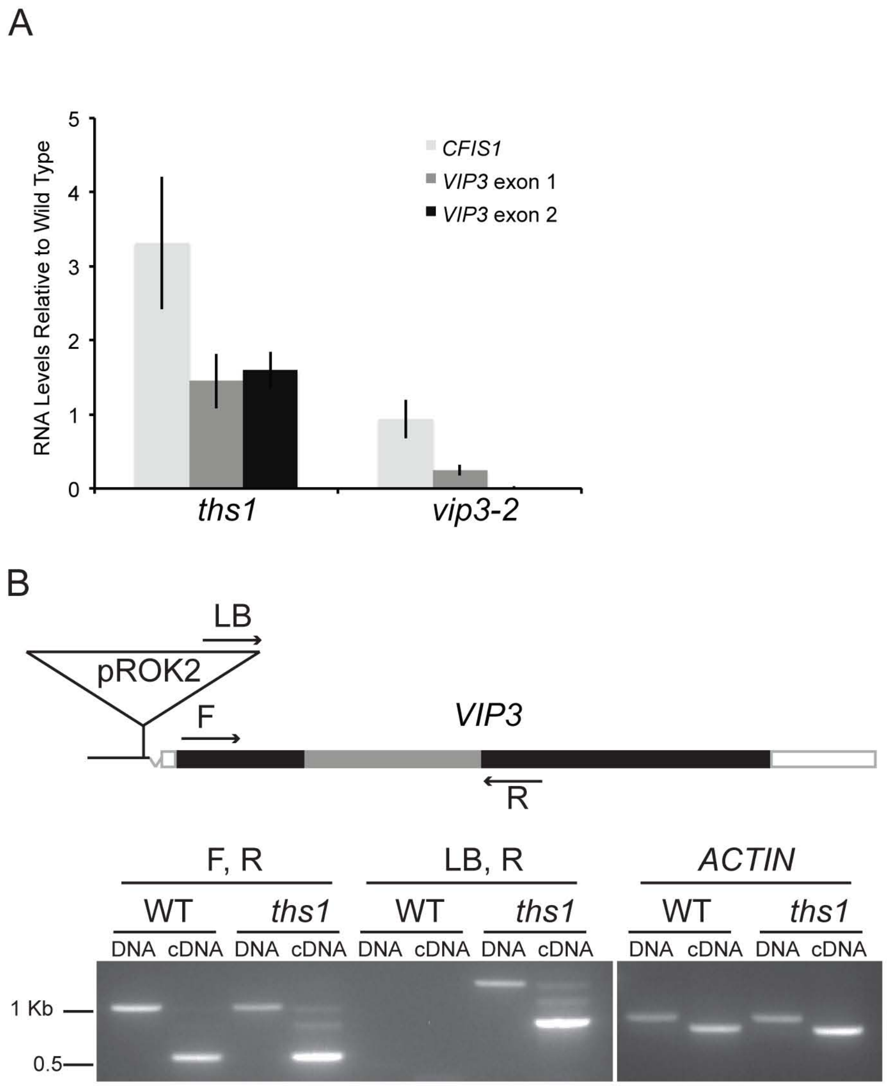

Supplementary Fig. S3. Transcript levels of *VIP3* and *CSIF1* in the *ths1* background. (A) Quantitative reverse-transcriptase PCR (RT-PCR) was used to amplify the first exon of *VIP3*, the second exon of *VIP3*, or the first three exons of *CSIF1*, from cDNA made from leaf tissue collected from the indicated backgrounds. Results for mutants are presented compared to Col-*0*. Data from five independent experiments, each with 2–3 technical replicates, are presented. Error bars indicate SEM. The *ths1* mutant showed a modest 1.5 to 3-fold increase in *VIP3* and *CFIS1* transcript levels compared to wild type. (B) Top panel, schematic of the *VIP3* gene, T-DNA insertion and the locations of oligos used for RT-PCR. Bottom panel, agarose gel of RT-PCR products amplified from Col-*0* or *ths1* genomic DNA and cDNA templates. Semi-quantitative RT-PCR detected an abundant transcript stretching from the left border of the T-DNA to the second exon of *VIP3*, along with several larger less abundant transcripts.

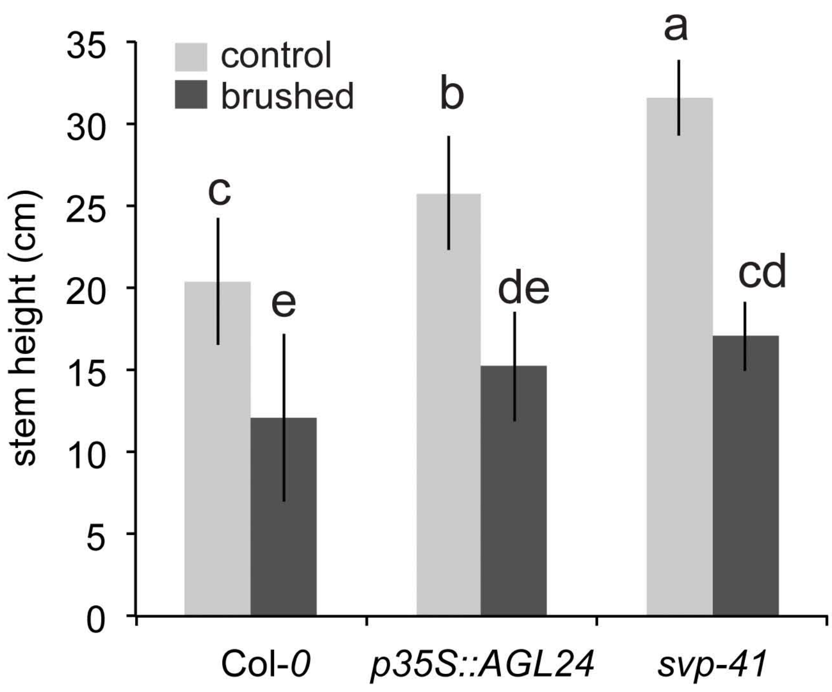

Supplementary Fig. S4. Two established early flowering lines are touch sensitive in the paintbrushing assay. At least 10 plants were used for each genotype and treatment. Error bars indicate standard deviation. Statistical groups represented with letters were determined by ANOVA followed by Scheffé's test, $P<0.05$. Plants were grown under 16 hours of light.

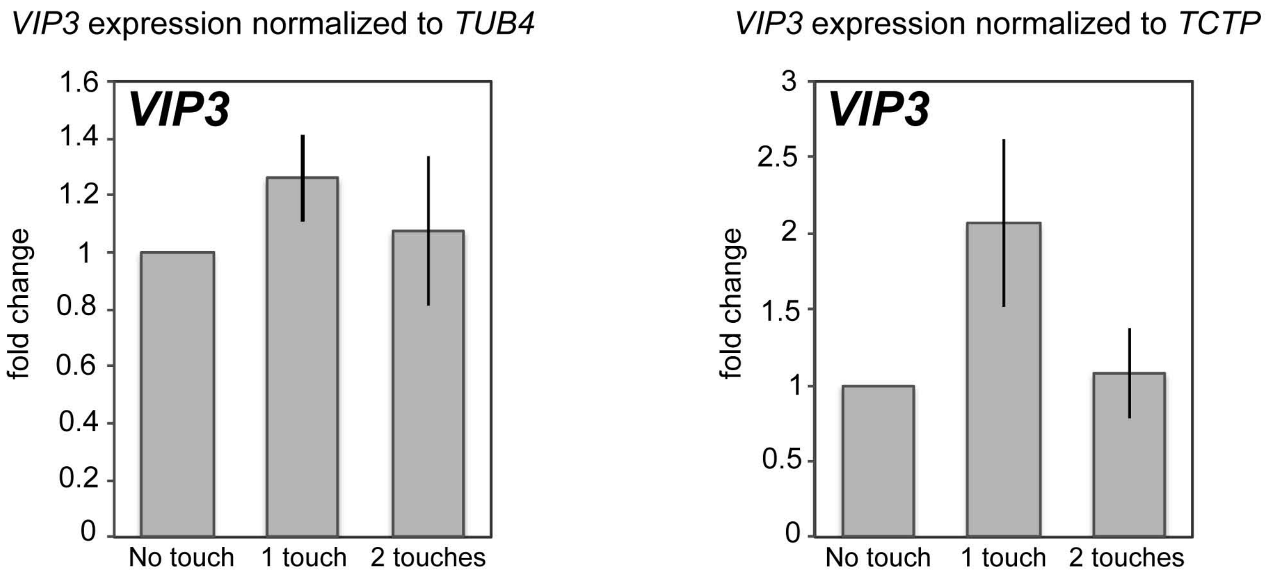

Supplementary Fig S5. *VIP3* transcript levels are not significantly affected by touch. Quantitative reverse-transcriptase PCR analysis of *VIP3* expression in wild type seedlings in response to touch. Twelve-day-old plants grown under long day conditions were brushed for 2 minutes with a paintbrush (as in Fig. 4C). RNA was extracted from the aerial tissues of treated and untreated plants and cDNA amplified with gene specific primers. Results were normalized to *TUB4* (left panel) or *TCTP* (right panel) and are presented as fold change compared to non-treated samples. The error bars indicate SEM from three replicates.

Supplementary Table S1. Oligos used in this study.

Name	Sequence	Application	Target	Figure
AD1	NTCGASTWTSGWGTT	TAIL-PCR	genomic DNA	
LBa1	TGGTTCACGTAGTGGGCCATCG	TAIL-PCR	genomic DNA	S2B
LBb1.3	ATTTTGCCGATTTCGGAAC	TAIL-PCR	genomic DNA	
LBb1	GCGTGGACCGCTTGCTGCAACT	TAIL-PCR/genotyping	genomic DNA	
29830.F	GCAATTAGCTGACGACGGCGGG	genotyping	*ths1*	
29830.R	GATCCAGCTCGTCCGGTCGCC	genotyping	*ths1*	
29830.F2	CTGACTGGATCTCTTGACGAGACG	genotyping	*vip3-2*	
29830.R2	GATACTCAGCAATTCCATATAGTACCCAAGC	genotyping	*vip3-2*	
29820.F	CTCATACCCGAAACACCCAC	genotyping	*cfis1-1*	
29820.R	GTGAAGAAGCTCGAGCGTTAGATATG	genotyping	*cfis1-1*	
SAIL.LB	GCCTTTTCAGAAATGGATAAATAGCCTTGCTTCC	genotyping	*cfis1-1*	
VIP5_062223.F	CTACACACAAGTACCTTAACGTCGTC	genotyping	*vip5-062223*	
VIP5_062223.R	CACCCGCTTTGAGACTAGCAG	genotyping	*vip5-062223*	
VIP6_065364_F	GATGCAACTGATGGGAAGGACTC	genotyping	*vip6-065364*	
VIP6_065364_R	CACCCATACATCAGGCATCTGAAG	genotyping	*vip6-065364*	
29820.F	CTCATAACCCGAAACACCCAC	RT-PCR	*CFIS1*	S1
29820.R	GTGAAGAAGCTCGAGCGTTAGATATG	RT-PCR	*CFIS1*	S1
ACT.F2	TACGCCAGTGGTCGTACAAC	RT-PCR	*ACTIN*	S1, S2B
ACT.R2	AACGACCTTAATCTTCATGCTGC	RT-PCR	*ACTIN*	S1, S2B
29820-QPCR-F	GAGGATGCTTGAACAACTCAAC	QRT-PCR	*CFIS1*	S2A
29820-QPCR-R	CAAAAGAAGCTCTTCGCGTCAAG	QRT-PCR	*CFIS1*	S2A
29830-QPCR-F	GAAACTCGCAGGTCTGAAATCG	QRT-PCR	*VIP3* exon 1	S2A
29830-QPCR-R	CATAACTTCACCGTCTCGTCAAG	QRT-PCR	*VIP3* exon 1	S2A

29830.QPCR.F3	GGTCTCCTAATGGGAAACGACTTG	QRT-PCR	*VIP3* exon 2	S2A
29830.QPCR.R3	CCTTACAGGCATATTGTGACCTTCTAG	QRT-PCR	*VIP3* exon 2	S2A
ACT.F-QPCR	CAACCGGTATTGTGCTGGATTC	QRT-PCR	*ACTIN*	S2A
Actin2.R-QPCR	GATGGCATGAGGAAGAGAGAAAC	QRT-PCR	*ACTIN*	S2A
Actin7.R-QPCR	GGATAGCATGAGGAAGAGCATAC	QRT-PCR	*ACTIN*	S2A
Actin8.R-QPCR	GAGACGGAGGATAGCATGTG	QRT-PCR	*ACTIN*	S2A

29830.F3	CTTGCTCACTTGAGGAAACACAG	RT-PCR	*VIP3*	S2B
29830-QPCR.R2	CCACCGAGAGAACGAATTTCTTG	RT-PCR	*VIP3*	S2B

TCTP QPCR-F	CATGCATACCCTCCCCAACAA	QRT-PCR	*TCTP*	4
TCTP QPCR-R	ACACCCAAGCTCAGCGAAGAA	QRT-PCR	*TCTP*	4
TUB4 QPCR-R	AGGGAAACGAAGACAGCAAG	QRT-PCR	*TUB4*	4
TUB4 QPCR-F	CTGTTTCCGTACCCTCAAGC	QRT-PCR	*TUB4*	4
TCH3 QPCR-F	TACCGTGATGTTTTCCCTCG	QRT-PCR	*TCH3*	4
TCH3 QPCR-R	GAGCTCATTCACGGTAATGTAACC	QRT-PCR	*TCH3*	4
TCH4 QPCR-F	CCATGTTGTTCCAGGTGATTTCAAG	QRT-PCR, ChIP-QPCR	*TCH4*	4
TCH4 QPCR-R	CCTCTGGTTCTGGATTCCAATC	QRT-PCR, ChIP-QPCR	*TCH4*	4
VIP3 QPCR-F	GTCCGTGTGTTTGATGTTGATAC	QRT-PCR	*VIP3*	S3
VIP3 QPCR-R	CTGCAACAGCAAGGATCGTAC	QRT-PCR	*VIP3*	S3
SAM ChIP QTF	TGAAGTCCAAAAGCAAAAACC	ChIP-QPCR	*SAM*	4
SAM ChIP QTF	GACGGAGAAGAAGAGCGAAA	ChIP-QPCR	*SAM*	4
TCH3ChIPQTF	GTGATGTTTTCCCTCGGTAA	ChIP-QPCR	*TCH3*	4
TCH3ChIPQTR	TTAGCGAGAAAGGGACGATG	ChIP-QPCR	*TCH3*	4
